# Inhibitory Activity of Quaternary Isoquinoline Alkaloids on Soluble Epoxide Hydrolase

**DOI:** 10.3390/cimb44090294

**Published:** 2022-09-16

**Authors:** Jang Hoon Kim, Chong Woon Cho, Mok Hur, Woo Tae Park, Youn-Ho Moon, Sung-Cheol Koo, Yun-Chan Hur, Jong Seong Kang, Ik Soo Lee

**Affiliations:** 1 Department of Herbal Crop Research, National Institute of Horticultural and Herbal Science, RDA, Eumseong 27709, Korea; 2 College of Pharmacy, Chungnam National University, Daejeon 34134, Korea; 3 Km Convergence Research Division, Korea Institute of Oriental Medicine, Daejeon 34134, Korea

**Keywords:** quaternary isoquinoline alkaloids, soluble epoxide hydrolase, non-competitive inhibitor, molecular simulation

## Abstract

The quaternary isoquinoline alkaloids of palmatine (**1**), berberine (**2**), and jatrorrhizine (**3**) were evaluated in terms of their ability to inhibit soluble epoxide hydrolase (sEH). They had similar inhibitory activities, with IC_50_ values of 29.6 ± 0.5, 33.4 ± 0.8, and 27.3 ± 0.4 μM, respectively. Their respective *K*i values of 26.9, 46.8, and 44.5 μM—determined by enzyme kinetics—indicated that they inhibited the catalytic reaction by binding noncompetitively with sEH. The application of computational chemistry to the in vitro results revealed the site of the receptor to which the ligand would likely bind. Accordingly, three alkaloids were identified as having a suitable basic skeleton for lead compound development of sEH inhibitors.

## 1. Introduction

Epoxyeicosatrienoic acids (EETs) produced by the epoxidation of olefinic bonds of arachidonic acid by the epoxygenase CYP enzymes exist in four regioisomeric forms, i.e., 5,6-EET, 8,9-EET, 11,12-EET, and 14,15-EET. They are endothelium-derived hyperpolarizing factors used to treat ischemic injury and have anti-inflammatory activity [1]. It was reported that 11,12-EET suppressed activity of TNF induced by NF-kB and the expression of vascular cell adhesion molecules in endothelial cells [1,2]. In mammals, soluble epoxide hydrolase (sEH), which belongs to the α/β-hydrolase family, is expressed in the cytoplasm of numerous tissues—such as adrenal, liver, kidney, lung, heart, and intestine [3]. sEH converts the epoxy group of EET into a diol to produce dihydroxyeicosatrienoic acid, which has poor anti-inflammatory effects [4]. Decreasing sEH activity increased the content of EETs in the cells, and efficacy against inflammation [5]. Moreover, the inhibition of sEH activity lowered blood pressure in animal models and confirmed the antihypertensive effect in clinical trials [6]. The inflammatory response that occurs during SARS-CoV-2 infection may lead to the expression of sEH in tissues [7]. sEH inhibitor and EETs have potential as inhibitors to alleviate the inflammatory response elicited by COVID-19 [8]. Potent urea-type inhibitors, such as 1-adamantanyl-3-(5-(2-(2-ethoxyethoxy)ethoxy)pentyl))urea (AUDA) and 1-trifluoromethoxyphenyl-3-(1-propionyl piperidin-4-yl)urea (TPPU), show inhibitory activity against sEH [9]. However, these inhibitors have several issues, such as low solubility and difficult formulation [10]. Consequently, new inhibitors are being sought via the synthesis of new compounds [11] and separation of components from natural products [12].

## 2. Materials and Methods

### 2.1. General Experimental Procedures

Palmatine chloride (SMB00472) and berberine chloride (B3251) were purchased from Sigma–Aldrich (St. Louis, MO, USA). Jatrorrhizine chloride (6681-15-8) was purchased from Aba Chem Scene (Monmouth Junction, NJ, USA). sEH (10011669), AUDA (479413-70-2), and 3-phenyl-cyano(6-methoxy-2-naphthalenyl)methyl ester-2-oxiraneacetic acid (PHOME; 10009134) were purchased from Cayman Chemical (Ann Arbor, MI, USA).

### 2.2. sEH Enzymatic Assay

The sEH assay was performed as described previously, with minor modifications [13]. To determine inhibitory activity, 130 μL of the sEH in 25 mM bis-Tris-HCl buffer (pH 7.0) containing 0.1% BSA was added to either 20 μL of inhibitor dissolved in MeOH. Next, 20 μL of PHOME was mixed to each mixture, which was then reacted at 37 °C for sEH hydrolysis. Product formation was monitored fluorometrically at 330 nm excitation and 465 nm emission for approximately 60 min.

sEH inhibition activity was calculated using

sEH Inhibition rate (%) = [(*Δ*C − *Δ*I)/*Δ*C] × 100 (1)

where *Δ*C and *Δ*I are the difference of the solvent and compound intensities, respectively, after about one hour and

y = y_0_ + (a × x/b + x) (2)

where y_0_ is the minimum value along the *y*-axis, a is the difference between the maximum and minimum values, and b is the x value at 50 percent of the “a” value.

### 2.3. Molecular Docking

Molecular docking was performed as described previously, with some modifications [14]. To dock the ligand into the receptor, three ligands having three-dimensional (3D) structures were minimized with MM2 charge using Chem3D Pro software (CambridgeSoft, Cambridge, MA, USA). The 3D structure of the sEH (3ANS) achieved from the RCSB protein data bank. Only the A-chain of this enzyme was necessary for docking, so the B-chain was not included. H_2_O and 4-cyano-N-[(1S,2R)-2-phenylcyclopropyl]benzamide were then excluded from the A-chain. The A-chain was added to hydrogen using AutoDockTools (Scripps Research, La Jolla, CA, USA); the Gasteiger charge model was then applied. Flexible ligand docking was achieved using a torsion tree, with detection of the torsion root and rotatable bonds. The grid box was set to a size of 126 × 126 × 126 at 0.375 Å. Molecular docking was achieved via a Lamarckian genetic algorithm with the maximum number of evaluations. The resulting values were calculated and presented using AutoDockTools, Chimera 1.14 (University of California, San Francisco, CA, USA), and LIGPLOT (European Bioinformatics Institute, Hinxton, UK) software.

### 2.4. Molecular Dynamics

Molecular dynamics (MD) was performed using the Gromacs 4.6.5 package [15]. The 3D structure of the ligand was built using the GlycoBioChem server. sEH Gro was produced using the GROMOS96 53a3 force field and a program database file (pdb). Their complex was surrounded by water molecules with six chloride (Cl^–^) anions. The energy minimization was stabilized to 10.0 kJ/mol using steepest descent minimization. The inhibitor–sEH complex was sequentially energy-minimized to constant temperature, constant volume (NVT) and constant temperature, and constant pressure (NPT) at 300 K using the particle mesh Ewald method with long-range electrostatics at 1 bar and MD simulation for 30 ns.

## 3. Results

### 3.1. Inhibition Effect of the Quaternary Isoquinoline Alkaloids on sEH

**Figure 1:**
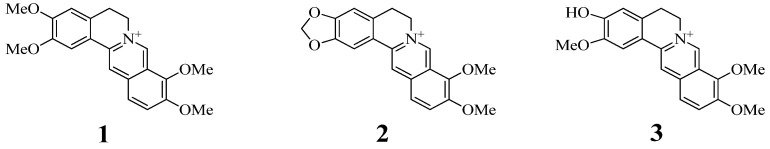
Structure of quaternary isoquinoline alkaloids (**1**–**3**).

Fluorometric determination (excitation wavelength, 330 nm; emission wavelength, 464 nm) was used to evaluate inhibitory activities toward sEH of the quaternary isoquinoline alkaloids palmatine (**1**), berberine (**2**), and jatrorrhizine (**3**) (Figure 1).

**Figure 2:**
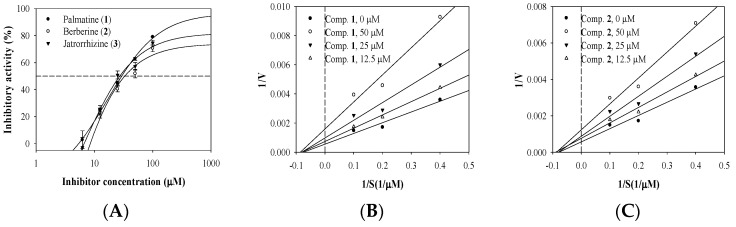

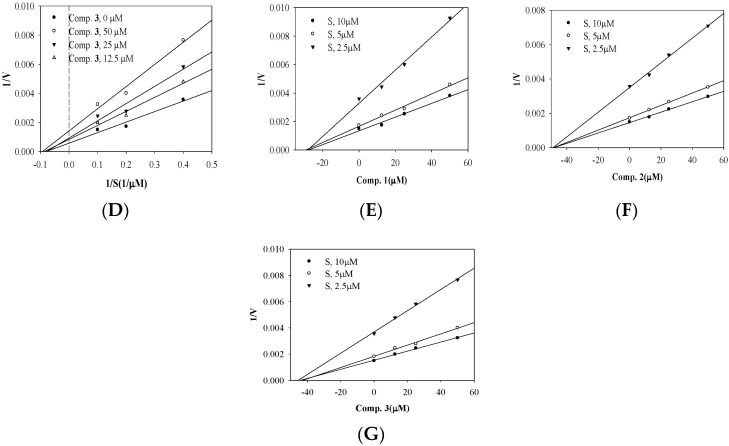
Inhibitory activity of compounds on sEH (**A**), Lineweaver–Burk (**B**–**D**), and Dixon (**E**–**G**) plots of sEH inhibition by compounds, respectively.

Inhibitory activity at a given concentration was calculated using Equation (1) in Materials and Methods and the inhibition rate of the compound at concentrations of 6.2–100 μM was derived from the IC_50_ value according to Equation (2) in Materials and Methods. The inhibitory activities of the three alkaloids (**1**–**3**) were thus calculated from their respective IC_50_ values of 29.6 ± 0.5, 33.4 ± 0.8, and 27.3 ± 0.4 μM in a dose-dependent manner. The positive control was AUDA (Figure 2A, Table 1).

### 3.2. Enzyme Kinetics

Additionally, an enzyme kinetic study was performed to identify how the three compounds bound to this enzyme. The initial velocity (*v*_0_) of inhibitor was determined as the rate of enzymatic conversion from substrate to product depending on the concentration of inhibitor at the start of the enzymatic reaction. The results were visualized using classic double-reciprocal Lineweaver–Burk plots. Figure 2B–D indicates one 1/*K*m and four 1/*V*max values for the three inhibitors, which inhibited the catalytic reaction of sEH with PHOME in a noncompetitive manner. Furthermore, Dixon plots showed that alkaloids (**1**–**3**) had inhibition constant (*k*_i_) values of 26.9, 46.8, and 44.5 μM, respectively (Figure 2E–G).

### 3.3. Molecular Docking Study

**Figure 3:**
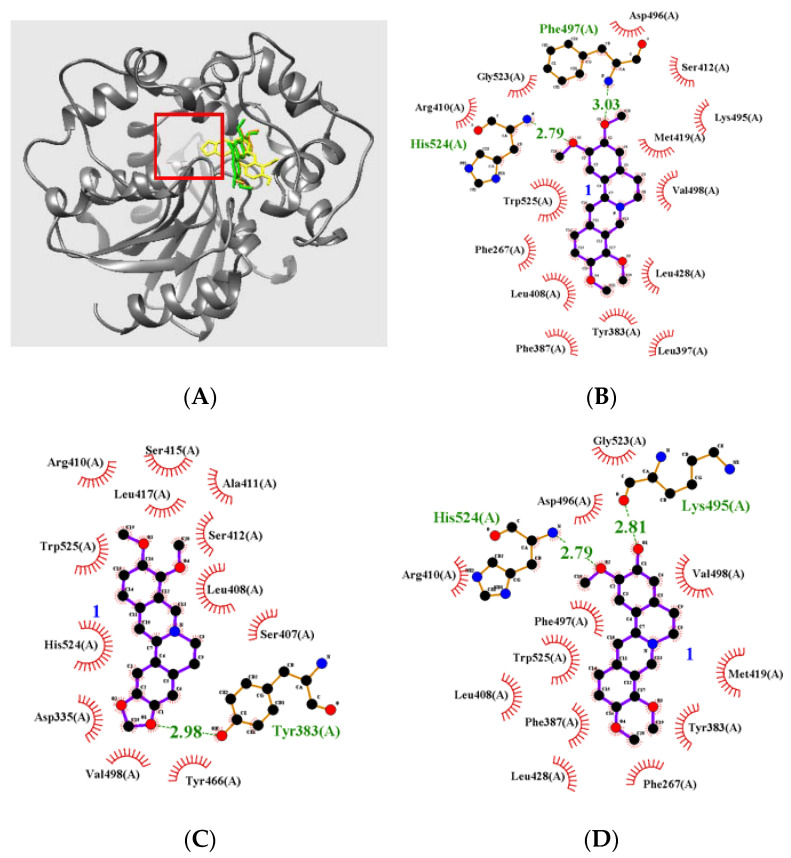
The binding pose (**A**) of three alkaloids **1**–**3** into sEH (red box: active site). Hydrogen bond interactions of inhibitors with the enzyme (**B**–**D**).

A molecular docking study based on computational chemistry was conducted to better understand the interaction between the amino acids of sEH and function groups of the quaternary isoquinoline alkaloids (**1**–**3**). The pdb files of sEH and the inhibitor were prepared and docking was simulated using the AutoDock ver. 4.2 package. The binding location between the three noncompetitively coupled inhibitors was identified via blind docking while setting the grid. Figure 3A and Table 2 showed that the three inhibitors (**1**–**3**) bound into the pocket surrounded by Pro379-Asn431, Glu494-Val500, and Cys522-Tr525 amino acids (left pocket) in sEH with similar AutoDock scores of −8.96, −9.14, and −8.82 kcal/mol, respectively (Figure 3A). Inhibitor **1** formed hydrogen bonds with Phe497 and His524 amino acids at distances of 3.03 and 2.79 Å, respectively. Inhibitor **2** was docked to interact with Tyr383 amino acid (2.98 Å). Inhibitor **3** formed hydrogen bonds with Lys495 and His524 amino acids at distances of 2.81 and 2.79 Å, respectively (Figure 3B–D).

### 3.4. Molecular Dynamics Study

**Figure 4:**
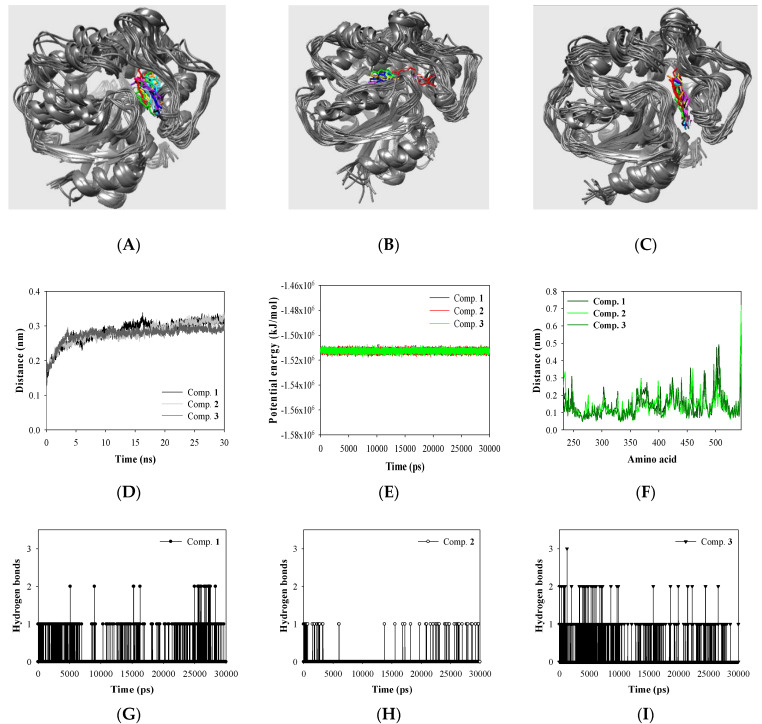
The superpositions of inhibitors **1**–**3** (**A**–**C**), the RMSD (**D**), potential energy (**E**), RMSF (**F**), hydrogen bonds (**G**–**I**) of the simulation calculated during 30,000 ps.

The electrostatic interactions of inhibitor with enzyme in the enzyme-inhibitor fluid state were determined through an MD study with the Gromacs 4.2 package running on the Linux operating system. Figure 4A–C shows that the virtual experiment yielded stable results. sEH modified the protein structure while maintaining about −1,510,000 kJ/mol potential energy within 3 Å root-mean-square deviation (RMSD) for inhibitors **1**–**3** for 20 ns (Figure 4D,E). The enzyme amino acids interacting with compounds **1** and **3** showed a similar root-mean-square fluctuation (RMSF) pattern, while those interacting with compound **2** showed a slightly different RMSF pattern. In particular, compound **1** exerted an effect on amino acids in the 500th range, leading to a change in RMSF value of ~4.5 Å. In contrast, compound **2** did not have a significant effect (Figure 4F). The number of hydrogen bonds increased in the order **2** < **1** < **3** (Figure 4G–I).

## 4. Discussion

MeOH and EtOH extracts of *Coptis chinensis* inhibited the catalytic reaction of sEH by 60.9% and 127.4%, respectively at a concentration of 25 µg/mL [16]. *Coptis chinensis* is one of the most popular traditional Chinese medicines; its main components—known as quaternary isoquinoline alkaloids—include jatrorrhizine, columbamine, coptisine, palmatine, and berberine [17]. Berberine is the major component, with a content of about 7% [17]. Berberine lowered the level of NOD-like receptor pyrin domain-containing protein 3 inflammasome and inhibited the epithelial-to-mesenchymal transition in diabetic rats [18]. Considerable research has been carried out to identify new compounds from medicinal plants to replace urea-type sEH-inhibitors [19,20].

Compounds of polyphenols and steroids had potential as sEH inhibitors [19,20]. Furthermore, *Berberis fremontii* [21], *Corydalis yanhusuo* [22], Annickia chlorantha, and *Annickia pilosa* [23] are representative as plants containing a lot of such quaternary alkaloids. 

However, a similar study of alkaloid inhibitors from natural plants has not been reported until now; this research established that the three quaternary isoquinoline alkaloids (**1**–**3**) have sufficient activity against sEH. Enzyme kinetics and molecular simulations confirmed that these inhibitors bind to the small pocket on the left (surrounded by Ser407-Phe429, Lys495-Val498, and Asp521-Thr526), rather than to the active site where substrates bind. However, synthetic inhibitors of the urea type were stably bound to the active site of sEH by computational chemistry [24]. Furthermore, protostane-type triterpenoids from *Alisma orientale* also bound to sEH with a low energy value by CDOCKER [15]. There was also a result that 4*H*-tomentosin, xanthalongin, and linoleic acid isolated from *Inula helenium* products can form a complex by bonding with Tyr343, ILe363, Tyr383, and His524 of the active sites [25].

The three compounds, which had similar inhibitory activity against the catalytic reaction with enzyme, differed only in the functional group (**1**: two methoxy, **2**: dioxol, **3**: a methoxy and a hydroxyl) of the A ring. Molecular dynamic experiments confirmed that this difference may affect the mechanism of binding between the ligand and receptor. In particular, inhibitor **2**—which was more rigid than compounds **1** and **3**—showed a tendency to maintain binding in the vacant space of the active site rather than relying on a fluid enzyme loop. Accordingly, the RMSF value of compound **2** displayed a relatively small change in the RMSF value compared with compounds **1** and **3**.

These findings suggest that the backbone can be divided into quaternary isoquinoline alkaloids and dioxol-quaternary isoquinoline alkaloids during the development of new sEH inhibitors.

## 5. Conclusions

Palmatine (**1**), berberine (**2**), and jatrorrhizine (**3**)—which are found in a medicinal plants—displayed inhibitory activity toward sEH in vitro with IC_50_ values of 29.6 ± 0.5, 33.4 ± 0.8, and 27.3 ± 0.4 μM, respectively. Enzyme kinetics studies and molecular simulations established that they inhibited catalytic reaction by binding to the left pocket (surrounded by Ser407-Phe429, Lys495-Val498, and Asp521-Thr526), of the active site of sEH as noncompetitive inhibitors. Isoquinoline quaternary alkaloids will play an important role in the development of new sEH inhibitors.

## Figures and Tables

**Table 1:** sEH inhibitory effect of quaternary isoquinoline alkaloids **1**–**3**.

Compound	Inhibition of Compounds on sEH ^a^	
	IC_50_ (μM)	Binding Mode (*k*_i_, M)
**1**	29.6 ± 0.5	Non-competitive(26.9)
**2**	33.4 ± 0.8	Non-competitive(46.8)
**3**	27.3 ± 0.4	Non-competitive(44.5)
AUDA ^b^	4.0 ± 1.3 nM	

^a^ Compounds were tested three times. ^b^ Positive control.

**Table 2:** Interaction of quaternary isoquinoline alkaloids **1–3** and autodock score for sEH.

	Autodock Score (kcal/mol)	Hydrogen Bonds (Å)
**1**	−8.96	Phe497(3.03), His524 (2.79)
**2**	−9.14	Tyr383 (2.98)
**3**	−8.82	Lys495 (2.81), His524 (2.79)

## Data Availability

Data are available upon request.
